# Early detection of microcirculatory changes using simultaneous heated and unheated transcutaneous blood gas monitoring: a proof-of-concept study in a porcine lipopolysaccharide model of systemic inflammation

**DOI:** 10.3389/fmed.2026.1859220

**Published:** 2026-09-01

**Authors:** Maud A. Hoofs, Louwrina H. te Nijenhuis, Norani H. Gangaram-Panday, Patricia A. C. Specht, Egbert G. Mik, Floor A. Harms, Irwin K. M. Reiss, Willem van Weteringen

**Affiliations:** 1 Department of Neonatal and Pediatric Intensive Care, Division of Neonatology, Erasmus MC Sophia Children’s Hospital, University Medical Center Rotterdam, Rotterdam, Netherlands; 2 Department of Cardiology, Thorax Center, Cardiovascular Institute, Erasmus MC, University Medical Center Rotterdam, Rotterdam, Netherlands; 3 Laboratory of Experimental Anesthesiology, Department of Anesthesiology, Erasmus MC, University Medical Center Rotterdam, Rotterdam, Netherlands; 4 Department of Pediatrics, Division of Neonatology and Pediatric Intensive Care, Children’s University Hospital Eppendorf, University Medical Center Hamburg-Eppendorf, Hamburg, Germany; 5 Department of Anesthesiology, Erasmus MC, University Medical Center Rotterdam, Rotterdam, Netherlands

**Keywords:** animal study, endotoxemia, lipopolysaccharide, microcirculation, non-invasive monitoring, sepsis, systemic inflammation, transcutaneous blood gas monitoring

## Abstract

**Introduction:**

Early detection of microcirculatory failure in septic patients has the potential to improve clinical outcomes. The microcirculation regulates the oxygen (O_2_) supply and carbon dioxide (CO_2_) washout in tissues and changes early during severe inflammation and sepsis. Transcutaneous blood gas sensors measure tissue O_2_ and CO_2_ levels at the skin surface, or when warmed use the vasodilatory response to approximate arterial levels. In addition to differences in measured O_2_ and CO_2_ values, systemic inflammation is likely to affect this vasodilatory effect of skin warming. This study investigated whether continuous comparison of heated and unheated transcutaneous blood gas monitoring could early detect microcirculatory changes using a porcine lipopolysaccharide (LPS) model of systemic inflammation.

**Methods:**

Thirty female Yorkshire x Norwegian Landrace pigs were subdivided into three study groups (control, LPS, and LPS with fluid resuscitation) and received LPS or saline control. Transcutaneous CO_2_ (tcPCO_2_) and O_2_ (tcPO_2_) were continuously and simultaneously measured using a heated (43 °C) and unheated (37 °C) transcutaneous sensor (Sentec OxiVenT™) at the porcine ear. Arterial blood gas measurements (PaCO_2_ and PaO_2_) were performed at baseline and 1, 2, and 3 h after LPS administration. At each time point, tcPCO_2_, tcPO_2_, and differences between transcutaneous and arterial blood gas measurements were determined. In addition, the unheated-heated tcPCO_2_ difference and the ratio between heated tcPCO_2_ and tcPO_2_ (tcPCO_2_/tcPO_2_) were calculated.

**Results:**

After LPS administration, a significant increase in heated tcPCO_2_ and a significant decrease in tcPO_2_ and tcPO_2_/tcPCO_2_ were observed. This resulted in a significant decrease in the unheated-heated tcPCO_2_ difference from 22.1 [18.8–33.2] to 0.7 [−8.3–8.8] mmHg in the LPS group, and from 23.0 [16.9–23.9] to −1.2 [−6.6–2.0] mmHg in the resuscitation group, while it remained 6.5 [3.1–9.8] mmHg in the control group. PaCO_2_ and PaO_2_ did not show large changes.

**Conclusion:**

Heated tcPCO_2_, tcPO_2_, unheated-heated tcPCO_2_, and tcPO_2_/tcPCO_2_ as observed using transcutaneous blood gas monitoring are promising parameters for early detection of microcirculatory changes during severe infections. In future studies, simultaneous heated and unheated transcutaneous measurements should be further explored in both healthy and septic patients.

## 1 Introduction

Sepsis is a life-threatening condition, resulting from a dysregulated response of the body to an infection (1). It can affect all patient groups, from premature neonates to the elderly, and it has been estimated that approximately 166 million sepsis cases occur worldwide each year, accompanied by high mortality rates (1–4). Sepsis in adults is diagnosed according to the sepsis-3 criteria if the patient has suspected or documented infection (based on body fluid cultures and antibiotics administration) and a 2-point increase of the Sequential Organ Failure Assessment (SOFA) score (based on vital signs and laboratory values) (1). However, these criteria may be insufficiently sensitive and could be late in identifying the onset of sepsis. In the hospital, close monitoring is of vital importance to detect the development of sepsis and initiate treatment as early as possible, as early intervention can improve patient outcomes (5–7).

Currently, most monitoring technologies in clinical care focus on the macrocirculatory status, despite the fact that the microcirculation plays a crucial role in the development and treatment of sepsis, septic shock, and other systemic inflammatory conditions (8–12). The microcirculation delivers oxygen (O_2_) to the tissues and enables washout of carbon dioxide (CO_2_). Under normal conditions, an increased O_2_ consumption and CO_2_ washout will lead to an increase in tissue perfusion to maintain adequate tissue O_2_ and CO_2_ levels. During shock, perfusion to the vital organs is maintained by a redistribution of blood flow from the peripheral microcirculation. An increased blood flow heterogeneity, reduced vessel density, and a lower proportion of perfused vessels have been observed in the microcirculation during sepsis, resulting in tissue hypoxia and hypercapnia (12–16). Close monitoring of CO_2_ and O_2_ levels in the skin microcirculation could, therefore, aid in the diagnosis and prognosis of sepsis or systemic inflammation (10, 11, 17, 18).

Transcutaneous blood gas monitoring is currently used to non-invasively and continuously monitor CO_2_ and O_2_ levels to assess the respiratory status of patients in (neonatal) intensive care units and surgical settings (19). Transcutaneous sensors use local skin heating to measure transcutaneous oxygen (tcPO_2_) and carbon dioxide (tcPCO_2_) values, which approximate arterial partial pressures of carbon dioxide (PaCO_2_) and oxygen (PaO_2_). Local heating causes arterialization of the skin, resulting in vasodilation and increased O_2_ and CO_2_ diffusion, which decrease the partial pressure gradient between blood and skin (19–21). During systemic inflammation, this arterialization effect is impaired due to the redistribution of blood, resulting in a large discrepancy between PaCO_2_ and tcPCO_2_ values (19, 22). While increased tcPCO_2_ and decreased tcPO_2_ levels might be indicative of both hemodynamic or respiratory insufficiency, poor agreement between transcutaneous and blood gas values has the potential for detection of microcirculatory impairment during a severe systemic inflammation. This discrepancy, however, necessitates frequent invasive blood gas withdrawals for verification and can also be caused by poor sensor placement.

**Figure 1:**
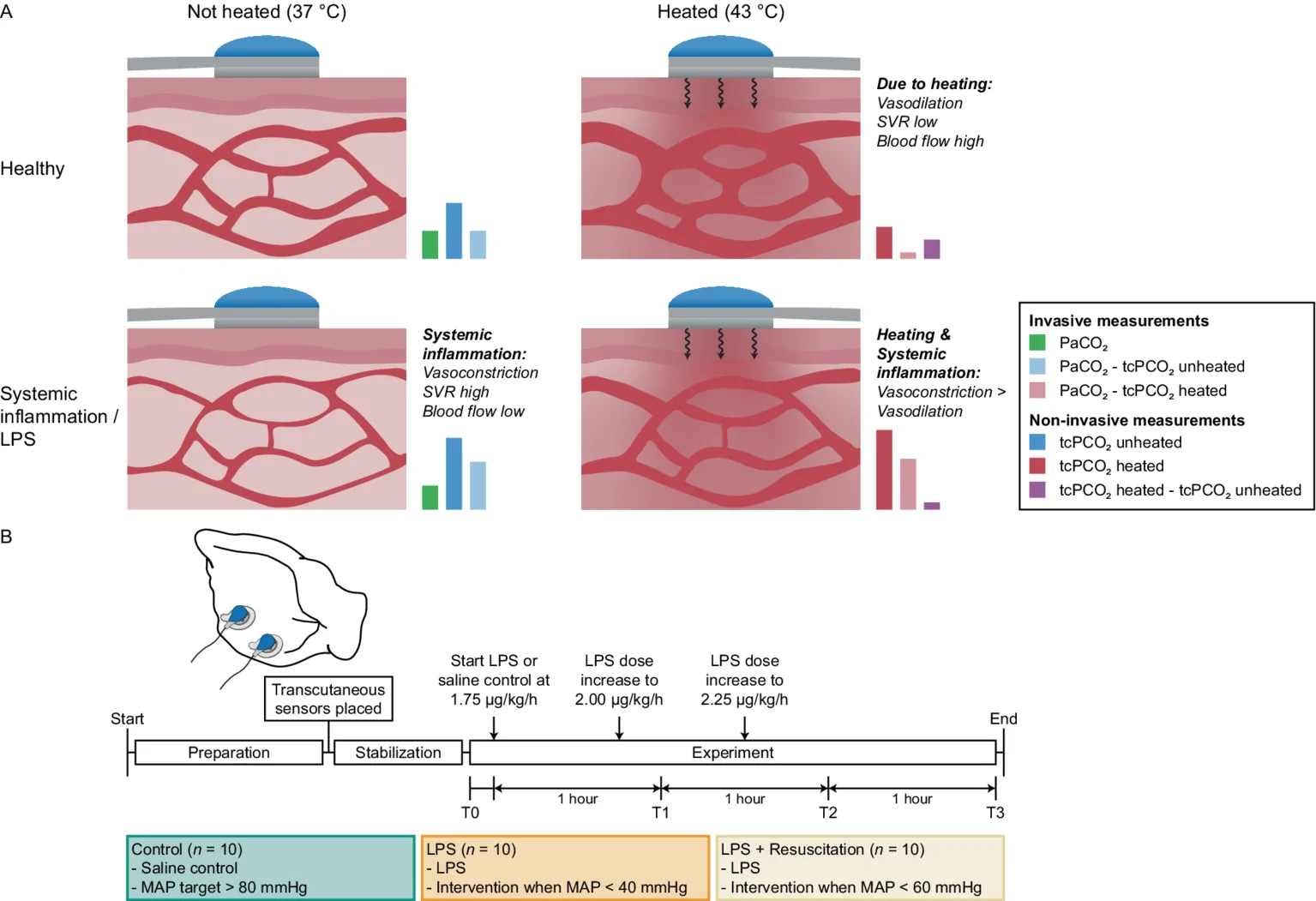
Study hypothesis and experimental protocol. **(A)** Effect of heating and systemic inflammation (LPS administration) on transcutaneous measurement values. **(B)** Experimental protocol. After the preparation phase two transcutaneous blood gas sensors (one heated and one unheated) were placed at the porcine ear. After a stabilization phase, the experiment was started. LPS, lipopolysaccharide; MAP, mean arterial pressure; *n*, number; PaCO_2_, arterial partial pressure of carbon dioxide; SVR, systemic vascular resistance; *T*, time point; tcPCO_2_, transcutaneous carbon dioxide.

An alternative approach to early detection of systemic inflammation could be the continuous monitoring of the effect of heating on the microcirculation to assess the patient’s hemodynamic state (18, 19, 23). This study aimed to determine the value of continuous simultaneous heated and unheated tcPCO_2_ measurements in a porcine lipopolysaccharide (LPS) model of severe systemic inflammation to capture changes in the skin microcirculation. Additionally, a comparison was made between changes in (1) tcPCO_2_ and PaCO_2_ levels, (2) tcPO_2_ and PaO_2_ levels, and (3) tcPO_2_ and tcPCO_2_ levels over time. We hypothesized that after LPS infusion, the difference between heated and unheated tcPCO_2_ decreases, while the difference between heated tcPCO_2_ and PaCO_2_ increases (Figure 1A).

## 2 Materials and methods

### 2.1 Study settings

In this animal study, 30 female Yorkshire x Norwegian Landrace pigs (3–4 months of age) were included. Pigs were chosen because transcutaneous blood gas measurements have shown a similar correlation between tcPCO_2_ and PaCO_2_ in these animals to the correlation seen in humans (24). In addition, the same medical equipment could be used, and the porcine epidermal thickness, skin vasculature, cardiac anatomy, physiology, and immune response have been shown to be similar to those in humans, making the porcine model of great value for the evaluation of new detection technologies during hemodynamic collapse (25–27).

All measurements were performed at the Laboratory of Experimental Anesthesiology, Erasmus MC, Rotterdam, The Netherlands. The study was performed in accordance with the Dutch Experiments on Animals Act and the ARRIVE guidelines (28). This study was part of a study protocol approved by the Central Authority for Scientific Procedures on Animals (license number AVD101002115658).

### 2.2 Animal preparation and maintenance

At the start of each experiment, the animals were sedated with an intramuscular injection of xylazine (2 mg/kg), tiletamine/zolazepam (6/6 mg/kg), and atropine sulfate (0.5 mg). After a 10-min resting period, pigs were intubated and mechanically ventilated using pressure-controlled ventilation (Maquet Servo-i Ventilator, Getinge AB, Rastatt, Germany). Intravenous access was obtained in an auricular vein, and anesthesia was induced using ketamine (100–300 mg) and tiletamine/zolazepam (50–100 mg). Three-lead electrocardiogram electrodes, an ear pulse oximeter, and a nasal temperature probe were placed for hemodynamic and respiratory monitoring. An arterial catheter (left femoral artery), central venous line (left femoral vein), thermodilution catheter (right femoral artery and right jugular vein, PiCCO_2_, Getinge AB, Gothenburg, Sweden), and indwelling urinary catheter were subsequently placed for blood collection, drug administration, hemodynamic assessment, and urine output measurement. As part of the main study protocol, surgical access was obtained to the kidney, liver, small intestines, and heart. A ureter catheter was surgically placed unilaterally. After all preparation steps, the pigs were placed on a warming pad in a lateral recumbent position and covered with a heating blanket (3 M™ Bair Hugger™ system, Saint Paul, Minnesota, United States) to maintain the body temperature at normal porcine levels (between 38 and 42 °C).

During the entire experiment, continuous anesthesia was maintained with ketamine (5 mg/kg/h), rocuronium bromide (4 mg/kg/h), midazolam (1.5 mg/kg/h), and sufentanil (4 μg/kg/h). Sodium chloride 0.9%, crystalloid (Sterofundin® ISO, B. Braun SE, Melsungen, Germany), and norepinephrine were continuously administered to maintain the desired mean arterial pressure (MAP) target as specified in the study protocol. At the start of the experiment, pigs received 500 mL colloid (Voluven®, Fresenius Kabi AG, Bad Homburg, Germany), 1,000 mg cefazolin (repeated after 4 h), and 1,000 mg magnesium sulfate. End-tidal CO_2_ (etCO_2_) was measured using capnography. Mechanical ventilation settings were adjusted to maintain normocapnia (etCO_2_ between 4 and 6 kPa) and normoxia (PaO_2_ between 10 and 16 kPa).

### 2.3 Study protocol

The complete study protocol is shown in Figure 1B. Pigs were consecutively assigned to one of three groups (controls, LPS, and LPS with fluid resuscitation), with 10 pigs per group. After animal preparation and a stabilization phase of at least 20 min to reach a minimal body temperature of 38 °C, baseline measurements (T0) were performed. After 1 h, LPS (*Escherichia Coli* O127: B8, L3880, Sigma-Aldrich, Saint-Louis, Missouri, United States) was administered at a rate of 1.75 μg/mL/kg. Every 45 min, the LPS dose was increased by 0.25 μg/mL/kg until the maximum dose of 2.25 μg/mL/kg was reached. Pigs in the control group received a vehicle control (sodium chloride 0.9%) at the same infusion volume and rate. Before LPS administration, the MAP was kept above 80 mmHg. After the start of LPS, this MAP target was changed to 40–60 mmHg in the LPS group and to 60–80 mmHg in the LPS with resuscitation group.

All measurements were repeated one, two, and three hours after LPS infusion (T1, T2, and T3). PaO_2_, PaCO_2_, pH, and lactate were determined (ABL Flex 800, Radiometer, Copenhagen, Denmark) from arterial and venous blood gas samples. Vital signs including heart rate (HR), peripheral oxygen saturation (SpO_2_), etCO_2_, and temperature were continuously logged at a 1 Hz sampling rate (Siemens SC 9000XL Monitor, Siemens-Elema, Solna, Sweden). Blood pressure was continuously monitored at a 1 Hz rate using the arterial catheter and PiCCO_2_ device, which was calibrated at each time point.

At the end of the experiments, the pigs were euthanized under general anesthesia with a lethal injection of 20 mL 15% potassium chloride. Animals were excluded and replaced in case of poor baseline conditions, septic conditions in the control group, non-septic conditions (no major change in MAP or lactate levels) in the LPS groups, or when experiments were terminated prematurely because humane endpoints were reached. More information about the study protocol, housing conditions, and humane endpoints is reported in a previous publication (29).

### 2.4 Transcutaneous measurements

Transcutaneous measurements were performed using two Sentec OxiVenT™ sensors (Sentec AG, Therwil, Switzerland) and two Sentec Digital Monitors (software versions 08.01.1–08.03.1 (Sentec Monitoring Board) and 06.01.00–06.03.01 (Multi Parameter Board), Sentec AG, Therwil, Switzerland). These sensors measure tcPO_2_ based on oxygen-dependent fluorescence quenching and contain a bicarbonate-buffered pH electrode to measure CO_2_ that diffuses from tissues (19, 21). The heating temperature was set to 43 °C at one (heated) sensor and to 37 °C at the other (unheated) sensor. Both sensors were placed on the same inner ear using attachment rings (MAR-MI, Sentec AG, Therwil, Switzerland), with at least 3 cm distance between them to prevent the heated sensor from arterializing the skin beneath the unheated sensor (Figure 1B). Contact gel (GEL-SD, Sentec AG, Therwil, Switzerland) was applied to the sensor before placement to promote gas diffusion and improve measurement accuracy. The ear was shaved and cleaned with ethanol 70% before sensor application and the sensors were calibrated using a reference gas according to the manufacturer’s guidelines. The automatic sensor heat reduction feature was switched off in order to maintain consistent heating during the entire experiment.

### 2.5 Transcutaneous parameters

Transcutaneous sensor parameters, including tcPO_2_, tcPCO_2_, temperature, and heating power, and sensor quality indicators were recorded with a sampling frequency of 1 Hz. HP describes the amount of power (in mW) needed to heat the sensor to the desired temperature.

In addition, novel parameters were calculated to quantify the differences in behavior between the macrocirculation and microcirculation. First, the difference and ratio between the heated and unheated transcutaneous CO_2_ measurements (*unheated-heated tcPCO_2_* difference and *unheated/heated tcPCO_2_* index) were determined to compare the changes in heated and unheated tcPCO_2_ values due to the changed microcirculation as a singular value. Similarly, the differences between the transcutaneous measurements and arterial blood gas values were determined (*tcPCO_2_-PaCO_2_* and *tcPO_2_-PaO_2_*) using the median transcutaneous values in the time window from 5 min before to 5 min after blood gas sampling. For the tcPCO_2_ measurement, both heated and unheated differences were determined. The difference between tcPCO_2_ and etCO_2_ values (*tcPCO_2_-etCO_2_*) was determined continuously, according to Vallée et al. (17). In addition, the ratios of the transcutaneous parameters to blood gas parameters (*tcPCO_2_/PaCO_2_* and *tcPO_2_/PaO_2_* index) and etCO_2_ (*etCO_2_/tcPCO_2_* index) were calculated. The final calculated parameter was the ratio between the heated tcPO_2_ and tcPCO_2_ values (*tcPO_2_/tcPCO_2_* index) to investigate the ratio between changes in transcutaneous O_2_ and CO_2_ values. The unheated tcPO_2_ values were not used in this study as without heating transcutaneous oxygen values approximate 0 mmHg (30). An overview of all transcutaneous parameters can be found in Supplementary Table S1.

Data points of tcPCO_2_, tcPO_2_, and heating power during device sensor alarms and notifications (for example ‘sensor off skin’ or ‘sensor stabilizing’) were removed. When the ‘sensor stabilizing’ notification occurred, all data points from the start of this error were removed until the moving average tcPCO_2_ slope (using a 30-s time window) became zero, in order to remove the arterialization period.

### 2.6 Statistical analysis

Statistical analyses were performed using MATLAB (version R2022b, The Mathworks, Inc., Natick, MA, USA) and R (version 4.4.2., R Foundation for Statistical Computing, Vienna, Austria). Median and interquartile ranges (IQR) parameters were determined per group (control, LPS, LPS with fluid resuscitation) and time point (T0, T1, T2, T3). For continuous parameters, the median value in the 10-min windows surrounding the blood gas sampling at each time point was determined. A Kruskal-Wallis test was performed to test differences between groups and a Friedman test was applied for differences between time points. In case of statistical significance, a Wilcoxon signed-rank test with Bonferroni correction was used. Data were visualized using boxplots. A *p*-value < 0.05 was considered statistically significant. The sample size (10 pigs per study group) was based on the primary outcome in the main investigation.

## 3 Results

### 3.1 Study characteristics

Measurements were performed in a total of 35 pigs, of which 5 were excluded due to poor baseline conditions (*n* = 1), not meeting group criteria (*n* = 3) or premature death (*n* = 1). The median weight of the pigs was 29.5 [28.2–31.4] kg and did not significantly differ between the study groups.

### 3.2 Heated and unheated tcPCO_2_

The unheated-heated tcPCO_2_ difference and unheated/heated tcPCO_2_ index decreased significantly over time in all study groups. The unheated-heated tcPCO_2_ difference became close to 0 mmHg in the LPS (0.7 [−8.3–8.8] mmHg) and resuscitation groups (−1.2 [−6.6–2.0] mmHg) (Supplementary Table S2 and Supplementary Figure S2).

**Figure 2:**
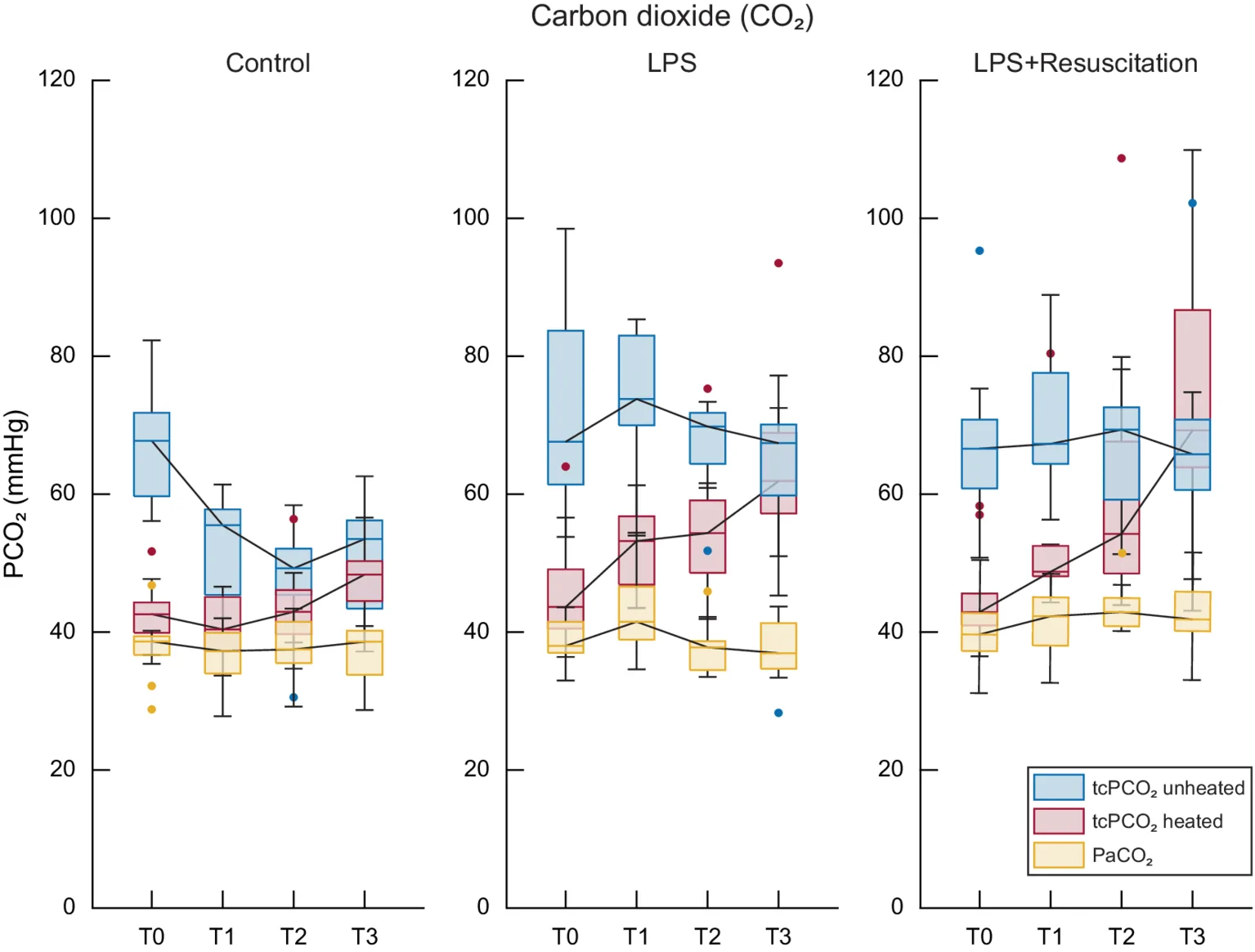
Boxplots of PaCO_2_ (yellow) and heated (red) and unheated (blue) tcPCO_2_ per study group. LPS, lipopolysaccharide; PaCO_2_, arterial partial pressure of carbon dioxide; *T*, time point; tcPCO_2_, transcutaneous carbon dioxide.

Figure 2 shows the boxplots of (heated and unheated) tcPCO_2_ and PaCO_2_ for the three study groups at each time point. In all groups, heated tcPCO_2_ levels were slightly higher than PaCO_2_ levels at T0. Heated tcPCO_2_ increased significantly over the course of the experiment in all study groups (from 42.6 [40.1–44.2] mmHg at T0 to 48.3 [45.4–49.9] mmHg at T3 (*p* = 0.01) in the control group, from 43.6 [40.8–48.4] mmHg to 61.9 [57.2–68.0] mmHg (*p* < 0.01) in the LPS group, and from 42.9 [41.1–45.6] mmHg to 69.2 [63.9–83.0] mmHg (*p* < 0.01) in the resuscitation group). Unheated tcPCO_2_ showed a significant decrease over time in the control group, although no significant decreases were found between individual time points. PaCO_2_ and etCO_2_ did not show large changes (Supplementary Table S2).

In the LPS groups, heated tcPCO_2_ deviated from the PaCO_2_ values toward the values of the unheated tcPCO_2_ at T3, resulting in a significant increase in heated tcPCO_2_-PaCO_2_ and tcPCO_2_/PaCO_2_ (all *p* < 0.01), as shown in Supplementary Figure S1. In the control group, heated tcPCO_2_-PaCO_2_ increased significantly between T1 and T3 (*p* < 0.01). All median parameter values per study group can be found in Supplementary Table S2.

### 3.3 TcPO_2_

**Figure 3:**
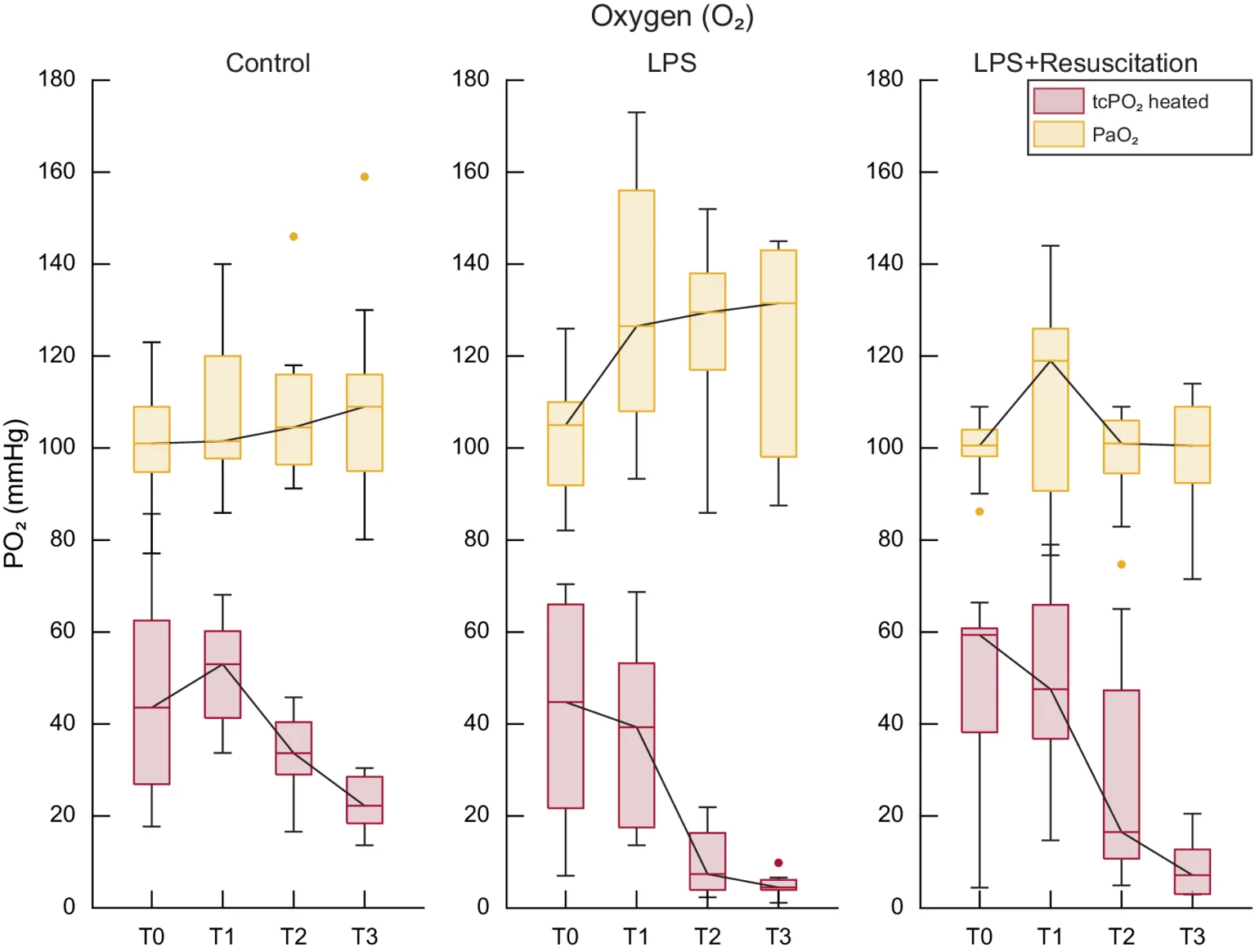
Boxplots of PaO_2_ (yellow) and heated (red) tcPO_2_ per study group. LPS, lipopolysaccharide; PaO_2_, arterial partial pressure of oxygen; *T*, time point; tcPO_2_, transcutaneous oxygen.

Whereas the PaO_2_ and SpO_2_ remained constant for all groups, the heated tcPO_2_ values decreased to values below 10 mmHg in the LPS groups at T3 (Figure 3). TcPO_2_ decreased significantly from 43.5 [28.7–58.5] mmHg at T0 to 22.2 [18.7–27.4] mmHg at T3 (*p* < 0.01) in the control group and from 59.3 [40.7–60.7] mmHg to 7.1 [3.4–12.1] mmHg (*p* < 0.01) in the resuscitation group. The LPS group already showed a significant decrease at T2 (from 44.8 [26.7–63.4] mmHg to 4.4 [4.0–5.9] mmHg; *p* < 0.01).

TcPO_2_-PaO_2_ showed the largest change from T0 to T3 in the LPS group (from −51.0 [−81.0 to −39.8] mmHg to −126.0 [−136.0 to −94.6] mmHg) and was significantly lower than the control group at T3 (−84.1 [−90.6 to −71.4] mmHg, *p* = 0.02). TcPO_2_/PaO_2_ became close to 0 in the LPS (0.04 [0.04 to 0.05]) and resuscitation groups (0.08 [0.03 to 0.13]) and was significantly lower in these groups compared to the controls (0.22 [0.18 to 0.27], *p* < 0.01) at T3. Supplementary Table S3 and Supplementary Figure S3 show the median values of the oxygen parameters per study group.

### 3.4 tcPO_2_/tcPCO_2_ index

The tcPO_2_/tcPCO_2_ index decreased significantly over time in all study groups and showed to be significantly lower in the LPS group at T1, T2, and T3 compared to the control group (Supplementary Table S4 and Supplementary Figure S2).

### 3.5 Other hemodynamic parameters

MAP was stable for the controls and showed a significant drop for the LPS and resuscitation group from 84.2 [80.3–92.4] to 60.6 [57.6–70.2] mmHg and from 84.7 [80.9–92.0] to 70.4 [66.2–73.0] mmHg, respectively (both *p* < 0.01, Supplementary Figure S4 and Supplementary Table S5). Lactate decreased significantly over time in the control group from 1.1 [0.9–1.3] to 0.7 [0.6–0.8] mmol/L (*p* < 0.01) and increased in the resuscitation group from 1.0 [0.8–1.4] to 1.8 [1.6–2.0] mmol/L (*p* < 0.01). Heating power levels did not change significantly over time (Supplementary Figure S5 and Supplementary Table S4).

## 4 Discussion

In this animal model of LPS-induced systemic inflammation, a decrease in the unheated-heated tcPCO_2_ difference was observed after LPS administration. Heated tcPCO_2_ levels increased from PaCO_2_ levels toward unheated tcPCO_2_ levels. The observed decrease in heated tcPO_2_ levels was less pronounced and observed later in the control group than in the LPS groups.

### 4.1 Transcutaneous PO_2_ and PCO_2_ monitoring

Transcutaneous monitoring of PO_2_ and PCO_2_ represents a promising non-invasive and continuous method for detecting microcirculatory changes in the skin during severe systemic inflammation. It uses local skin warming to achieve skin arterialization, resulting in tcPO_2_ and tcPCO_2_ values that approximate the gold standards PaO_2_ and PaCO_2_ (31). The achieved vasodilation reaches a plateau at skin temperatures of approximately 42 °C, ensuring comparable and reproducible measurements (32, 33). When local heating is not applied, tissue PO_2_ and PCO_2_ levels can be monitored to provide additional information on skin microcirculation.

A significant decrease in the unheated-heated tcPCO_2_ difference and unheated/heated tcPCO_2_ index was observed in the LPS groups. Both parameters were found to be lower in the LPS group when compared to controls, reflecting the inability of the microcirculation to increase blood flow during LPS-induced endotoxemia, resulting in tcPCO_2_ levels similar to the values of unheated tissue. Orbegozo et al. (23) showed a lower ratio between heated and unheated blood flow in septic shock non-survivors, suggesting a smaller effect of heating on blood flow during septic shock. Vallée et al. (18) showed a less evident tcPCO_2_ decrease due to heating in septic patients when compared to healthy controls. In that study skin temperature was increased from 37 to 45 °C at a single moment and could not be repeated at the same location due to the remaining arterialization effect after switching off the heating. Where previous studies used a heating challenge to assess microvascular reactivity, the unique aspect of this study is that heated and unheated tcPCO_2_ measurements were performed simultaneously, allowing continuous and real-time assessment of the microcirculatory state.

### 4.2 The LPS model

Administration of LPS, a component of the cell membrane of Gram-negative bacteria, induces an inflammatory response in humans and animals. This reaction is characterized by an increase in immune cells and interleukins, and septic symptoms such as the hypotension and hyperlactatemia observed in our LPS model (34–36). The heterogenous blood flow distribution of the microcirculation during systemic inflammation can result in insufficient supply of O_2_ and accumulation of CO_2_ at skin level, explaining the decrease in tcPO_2_ levels and increase in tcPCO_2_ levels shown in our study. Previous studies using LPS models in dogs and rats showed a decreased muscle and mitochondrial PO_2_ and an increased subcutaneous and gut luminal PCO_2_ (37–39). Similarly, a decreased tcPO_2_ and an increased tcPCO_2_ have been demonstrated in septic humans, which was worse in non-survivors (40–42). However, respiratory function should always be accounted for.

### 4.3 Transcutaneous PO_2_ and PCO_2_ and blood gas measurements

Differences and ratios between transcutaneous and blood gas measurements were used as an indicator of microcirculatory changes in the skin during LPS-induced endotoxemia, adjusted for the effect of respiratory deterioration on transcutaneous blood gas values. A decline in both blood gas and transcutaneous measurements suggests a poor respiratory function, while a substantial discrepancy between transcutaneous and blood gas values suggests a change in skin microcirculation due to a shift in blood supply to the vital organs. The observed deviation of heated tcPO_2_ and tcPCO_2_ from blood gas values, tcPO_2_/PaO_2_ decrease toward 0, and tcPCO_2_/PaCO_2_ increase to values above 1 in the LPS groups, suggest a relatively lower tcPO_2_ and higher tcPCO_2_. Vallée et al. (17) showed a decreased tcPCO_2_-PaCO_2_ when blood flow is increased. In addition, an increased tcPCO_2_-PaCO_2_ and tcPO_2_-PaO_2_ have been observed in neonates and adults with sepsis, shock, high lactate or a lower cardiac index (17, 18, 21, 22, 31, 40, 43, 44). The large tcPO_2_-PaO_2_ (between −40 and −60 mmHg) difference already at baseline should be noted. This large offset could reflect insufficient heating of the thicker porcine skin or improper sensor application. In addition, the Severinghaus correction used to estimate arterial CO_2_ values using measurements at human skin, might not be optimal for measurements at the thicker skin. Despite these baseline differences, the transcutaneous sensor was able to observe significant microcirculatory changes following LPS infusion. This suggests that tcPO_2_-PaO_2_ might also be of diagnostic value in adults or animals with a thicker epidermis, although invasive blood gas withdrawal is still needed to determine this parameter.

In addition to the observed differences between study groups, tcPO_2,_ heated tcPCO_2_-PaCO_2_, and unheated tcPCO_2_ also deteriorated in the control group over time. This general deterioration could reflect the effects of prolonged anesthesia and surgical exposure. The lower tcPO_2_ and higher heated tcPCO_2_-PaCO_2_ can be explained by the increased skin metabolism due to skin heating, thereby increasing the O_2_ consumption and CO_2_ production. The decrease in unheated tcPCO_2_ suggests an improved blood flow with subsequent CO_2_ tissue washout, and is most probably the effect of the protocolized fluid administration from the start of the experiments.

### 4.4 Other transcutaneous blood gas parameters

Besides the simultaneous heated and unheated measurements, several other noninvasive alternatives to invasive blood gas withdrawals are available, including etCO_2_ measurements. Our results showed significant differences in heated tcPCO_2_-etCO_2_ and heated tcPCO_2_/etCO_2_ between study groups after LPS administration. These differences were present even when using the unheated transcutaneous measurements and are in accordance with the results of Vallée et al. (17), showing a larger tcPCO_2_-etCO_2_ difference in septic shock patients. However, replacing blood gas withdrawals with etCO_2_ is only possible in mechanically ventilated patients, while severe systemic inflammation and sepsis are ideally diagnosed early and before mechanical ventilation is needed. In addition, large differences may also result from other factors such as skin thickness and poor sensor placement.

The tcPO_2_/tcPCO_2_ index decreased in all study groups, but approached 0 in the LPS groups. An index smaller than 1 indicates that the increasing tcPCO_2_ exceeds the decreasing tcPO_2_, which could be a sign of a critical hypoxic condition. Tatevossian et al. (42) observed lower values in non-survivors of septic shock when compared to survivors, and showed an increased mortality for an index below 1. The tcPO_2_/tcPCO_2_ index shows potential for observing deterioration in critically ill patients.

Heating power, required to reach the set sensor temperature, did not show any differences between controls and pigs receiving LPS and showed no changes over time. Although a reduced heat dissipation could be expected during hypoperfusion, lowering the required heating power to maintain set temperatures, heating power could also be influenced by the macrocirculatory status, core temperature, and ambient temperature. The exact effects of these factors on heating power should first be identified before the added value of this parameter to assess the hemodynamic status of septic patients can be validated.

### 4.5 Limitations

The use of the LPS model could be challenged. The observed changes in the LPS groups might reflect both macrocirculatory and microcirculatory effects, as MAP dropped significantly in these groups. However, changes in microcirculation were also observed in this model using the microcirculatory monitoring technology dynamic light scattering (29). The presence of a severe inflammatory response was not supported by molecular inflammatory biomarkers such as interleukins, C-reactive protein (CRP) or procalcitonin (PCT) as these were not determined during the conduct of this study. Although systemic inflammatory response symptoms were observed after LPS administration, caution should be applied with any conclusions derived from the LPS model, and the new transcutaneous parameters should be investigated in septic patients to prove their clinical value. Another study limitation was the relatively short observation time. Measurements were performed during the 3 hours following LPS administration to observe early changes in the microcirculation, while various physiological mechanisms appear several hours after onset of systemic inflammation or sepsis. In addition, the difference between the heated and unheated transcutaneous measurements could have been influenced by differences in body temperature between pigs. Although a relative hypothermia could arise due to the temperature loss during the preparation steps, any hypothermic effect was most probably eliminated by the standard temperature of 37 °C of the unheated sensor and the stabilization phase of at least 20 min before LPS administration. Endotoxemia-induced hyperthermia could affect the transcutaneous measurement values due to increased skin temperature, cutaneous perfusion, gas diffusion, and arterialization. It is still expected that during endotoxemia, the microcirculatory response to either LPS-induced hyperthermia or to the additional heating is less than in healthy subjects, resulting in heated tcPCO_2_ values approaching unheated tcPCO_2_ values. In this study, no hyperthermia was observed as sensor temperatures never exceeded the set temperature. The ethanol used to clean the skin before sensor placement could have influenced the skin microcirculation. Langer et al. (45) showed a decrease in functional capillary density and red blood cell velocity after application of ethanol. However, as it was applied to all pigs, it could not explain differences between the study groups. Another limitation is the small sample size of each study group. As ideally both the effect of the differences between groups and the effect of time since LPS administration on the transcutaneous measurements would be evaluated, a mixed-effects model would be the preferred statistical method. However, due to the small number of blood gas withdrawals, only a small number of data points would be available for a model. Another limitation is that both transcutaneous measurements were performed at the same ear to maximize microcirculation comparability. Although at least 3 cm separated the two sensors, the unheated sensor could be influenced by the heating of the other sensor (46). The ear is one of the most well-perfused and heat dissipating regions of the body and it is not known whether the same observations would be made at other skin sites.

### 4.6 Future perspectives

The simultaneous use of a heated and unheated transcutaneous sensor could offer real-time assessment of the microcirculatory status without the influence of the respiratory condition of the patient and without invasive blood gas withdrawal. In future studies, the parameters evaluated in this study should be measured in adults and neonates at risk for sepsis development and compared to healthy controls. The challenge for future studies is to combine these transcutaneous parameters with biomarkers for infection and inflammation, such as CRP, PCT, interleukins, and lactate, and other non-invasive continuous measurements to develop an optimal sepsis detection model which is able to diagnose sepsis in an early stage with high sensitivity and specificity, differentiate sepsis from other types of shock and to determine the sepsis severity.

## 5 Conclusion

Simultaneous heated and unheated transcutaneous blood gas monitoring revealed a decreasing unheated-heated tcPCO_2_ difference and a declining tcPO_2_ and tcPO_2_/tcPCO_2_ index in pigs receiving LPS, indicating microcirculatory compromise. The non-invasive nature and ability to both reflect respiratory and hemodynamic state make this measurement method a promising monitoring tool for early detection of microcirculatory changes during severe hemodynamic conditions as systemic inflammation, sepsis, and shock.

## Data Availability

The original contributions presented in the study are included in the article/Supplementary material, further inquiries can be directed to the corresponding author.
