# Supplementary material for: Early detection of microcirculatory changes using simultaneous heated and unheated transcutaneous blood gas monitoring: a proof-of-concept study in a porcine lipopolysaccharide model of systemic inflammation
Source: Front Med (Lausanne). 2026 Sep 1;13:1859220. doi: 10.3389/fmed.2026.1859220 (PMC13574667; doi:10.3389/fmed.2026.1859220)
Supplement: Supplementary file 1 [file Data_Sheet_1.pdf]

Supplementary Material

Contents

1 Supplementary Table S1 Transcutaneous parameters 2
2 Supplementary Figure S1 Boxplots CO_2 parameters 4
3 Supplementary Figure S2 Boxplots unheated vs heated parameters 5
4 Supplementary Figure S3 Boxplots O_2 parameters 6
5 Supplementary Figure S4 Boxplots MAP and lactate 7
6 Supplementary Figure S5 Boxplots heating power 8
7 Supplementary Table S2 CO_2 related parameters per study group 9
8 Supplementary Table S3 O_2 related parameters per study group 11
9 Supplementary Table S4 $TcPO_2$ / $TcPCO_2$ and heating power per study group 12
10 Supplementary Table S5 MAP and lactate per study group 13

1 **Supplementary Table S1** Transcutaneous parameters

CO_2 related parameters

**Parameter**	**Calculation/definition**	**Unit**
$tcPCO_2$ heated	$tcPCO_2$ determined with heated sensor	mmHg
$tcPCO_2$ unheated	$tcPCO_2$ determined with unheated sensor	mmHg
$tcPCO_2$-$PaCO_2$ heated	$tcPCO_2heated - PaCO_2$	mmHg
$tcPCO_2$/$PaCO_2$ heated	$\frac{tcPCO_2heated}{PaCO_2}$	-
$tcPCO_2$-$etCO_2$ heated	$tcPCO_2heated - etCO_2$	mmHg
$etCO_2$/$tcPCO_2$ heated	$\frac{etCO_2}{tcPCO_2heated}$	-
$tcPCO_2$-$PaCO_2$ unheated	$tcPCO_2unheated - PaCO_2$	mmHg
$tcPCO_2$/$PaCO_2$ unheated	$\frac{tcPCO_2unheated}{PaCO_2}$	-
$tcPCO_2$-$etCO_2$ unheated	$tcPCO_2unheated - etCO_2$	mmHg
$etCO_2$/$tcPCO_2$ unheated	$\frac{etCO_2}{tcPCO_2unheated}$	-
unheated-heated $tcPCO_2$	$tcPCO_2unheated - tcPCO_2heated$	mmHg
unheated/heated $tcPCO_2$	$\frac{tcPCO_2unheated}{tcPCO_2heated}$	-

Parameters determined with the heated transcutaneous sensor are highlighted in red, the parameters of the unheated sensor are highlighted in blue. *CO_2* carbon dioxide, *$etCO_2$* end-tidal carbon dioxide, *$PaCO_2$* arterial partial pressure of carbon dioxide, *$tcPCO_2$* transcutaneous carbon dioxide

O_2 related parameters

**Parameter**	**Calculation/definition**	**Unit**
$tcPO_2$ heated	$tcPO_2$ determined with heated sensor	mmHg
$tcPO_2$-PaO_2 heated	$tcPO_2 heated - PaO_2$	mmHg
$tcPO_2/PaO_2$ heated	$\frac{tcPO_2 heated}{PaO_2}$	-

Parameters determined with the heated transcutaneous sensor are highlighted in red. *O_2* oxygen, *PaO_2* arterial partial pressure of oxygen, *$tcPO_2$* transcutaneous oxygen

Other parameters

**Parameter**	**Calculation/definition**	**Unit**
$tcPO_2$ / $tcPCO_2$ heated	$\frac{tcPO_2 heated}{tcPCO_2 heated}$	-
Heating power heated	power needed to obtain the desired temperature of the heated sensor	mW
Heating power unheated	power needed to obtain the desired temperature of the unheated sensor	mW

Parameters determined with the heated transcutaneous sensor are highlighted in red, the parameters of the unheated sensor are highlighted in blue. *$tcPCO_2$* transcutaneous carbon dioxide, *$tcPO_2$* transcutaneous oxygen

2 **Supplementary Figure S1** Boxplots CO_2 parameters

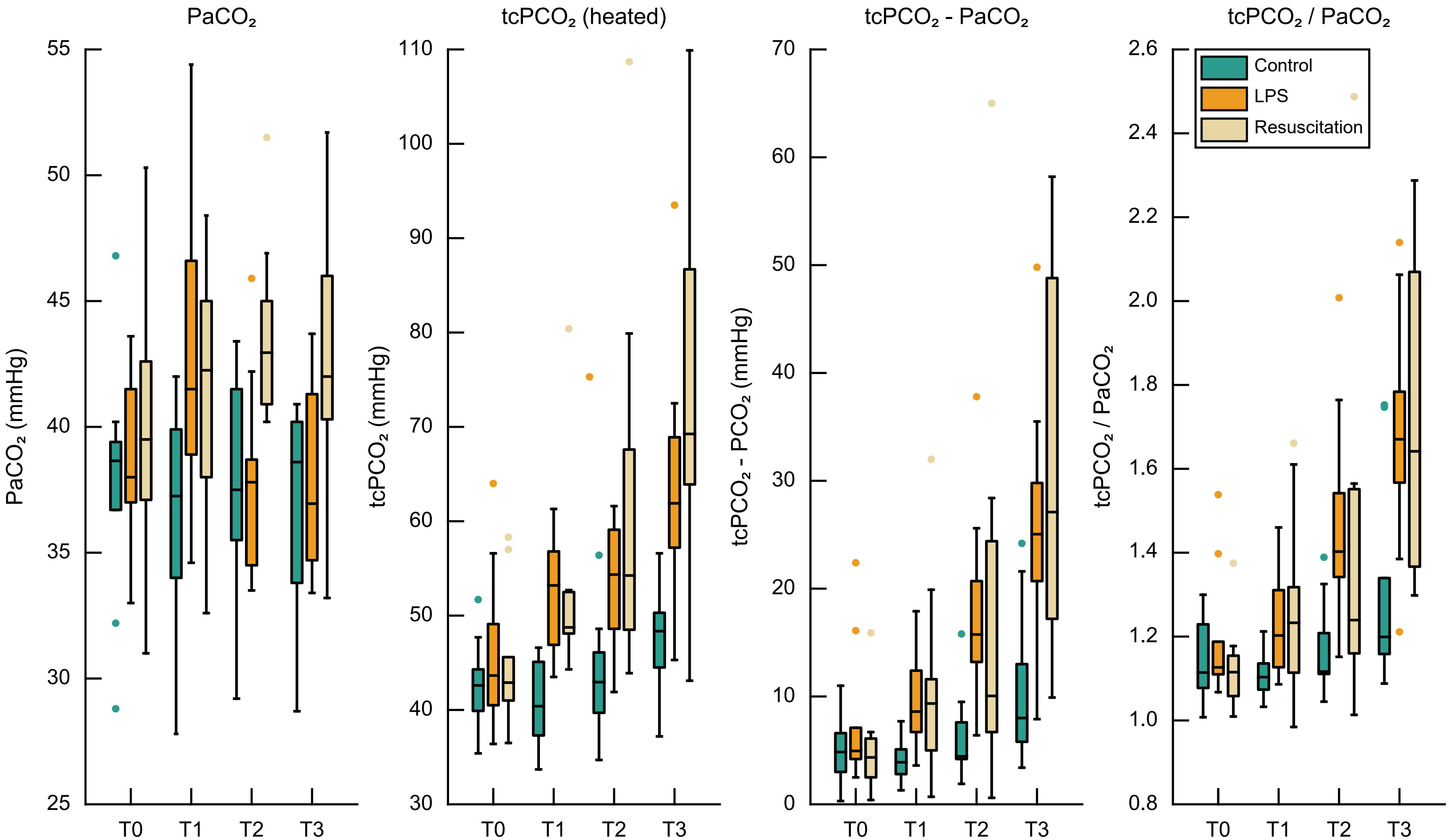

Figure S1 Boxplots of $PaCO_2$, $tcPCO_2$, $tcPCO_2$-$PaCO_2$, and $tcPCO_2/PaCO_2$ using the heated transcutaneous sensor per study group. *LPS* lipopolysaccharide, *$PaCO_2$* arterial partial pressure of carbon dioxide, *T* time point, *$tcPCO_2$* transcutaneous carbon dioxide.

3 **Supplementary Figure S2** Boxplots unheated vs heated parameters

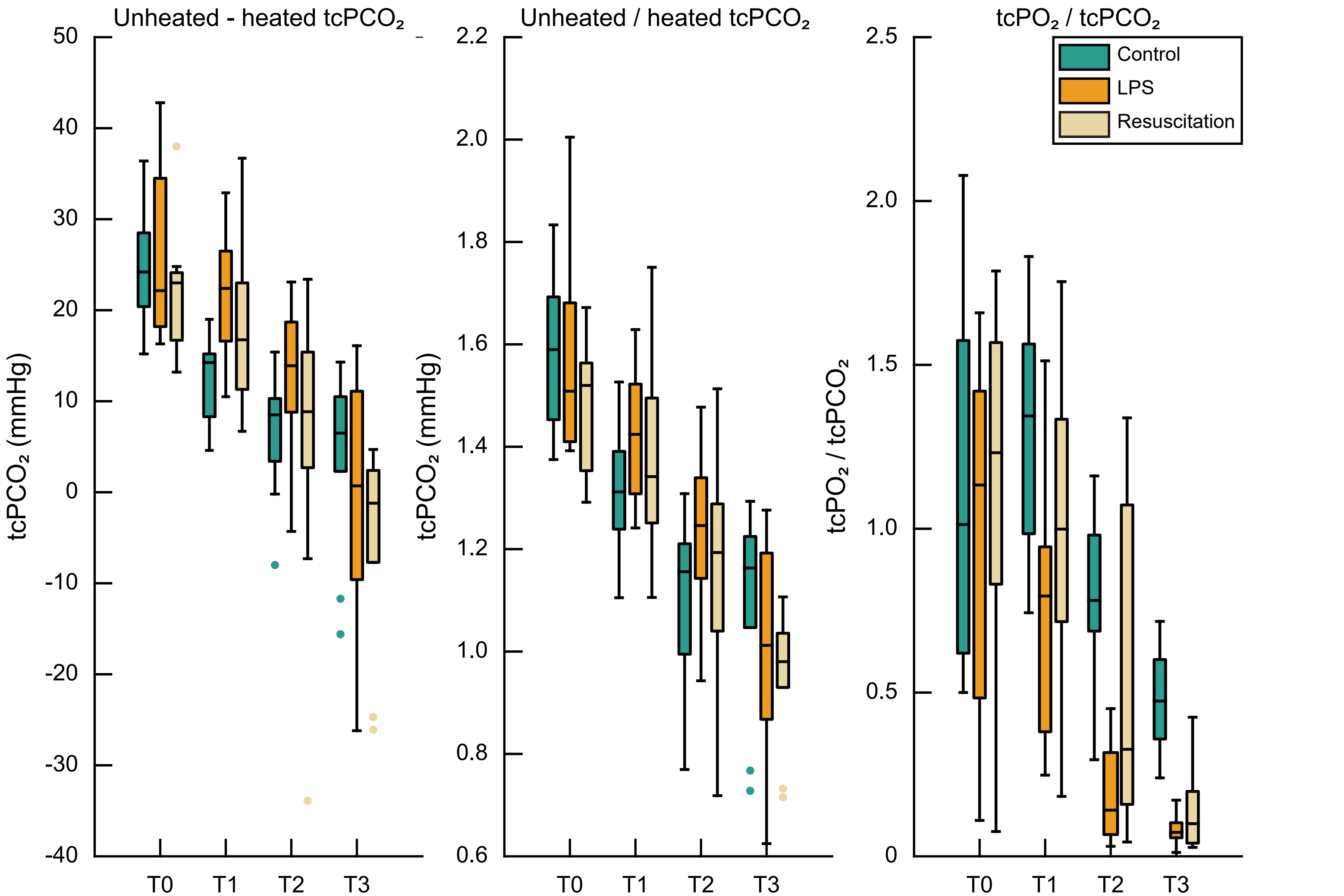

Figure S2 Boxplots of unheated-heated $tcPCO_2$, unheated/heated $tcPCO_2$, and $tcPO_2/tcPCO_2$ heated per study group. *LPS* lipopolysaccharide, *T* time point, *$tcPCO_2$* transcutaneous carbon dioxide, *$tcPO_2$* transcutaneous oxygen.

4 **Supplementary Figure S3** Boxplots O_2 parameters

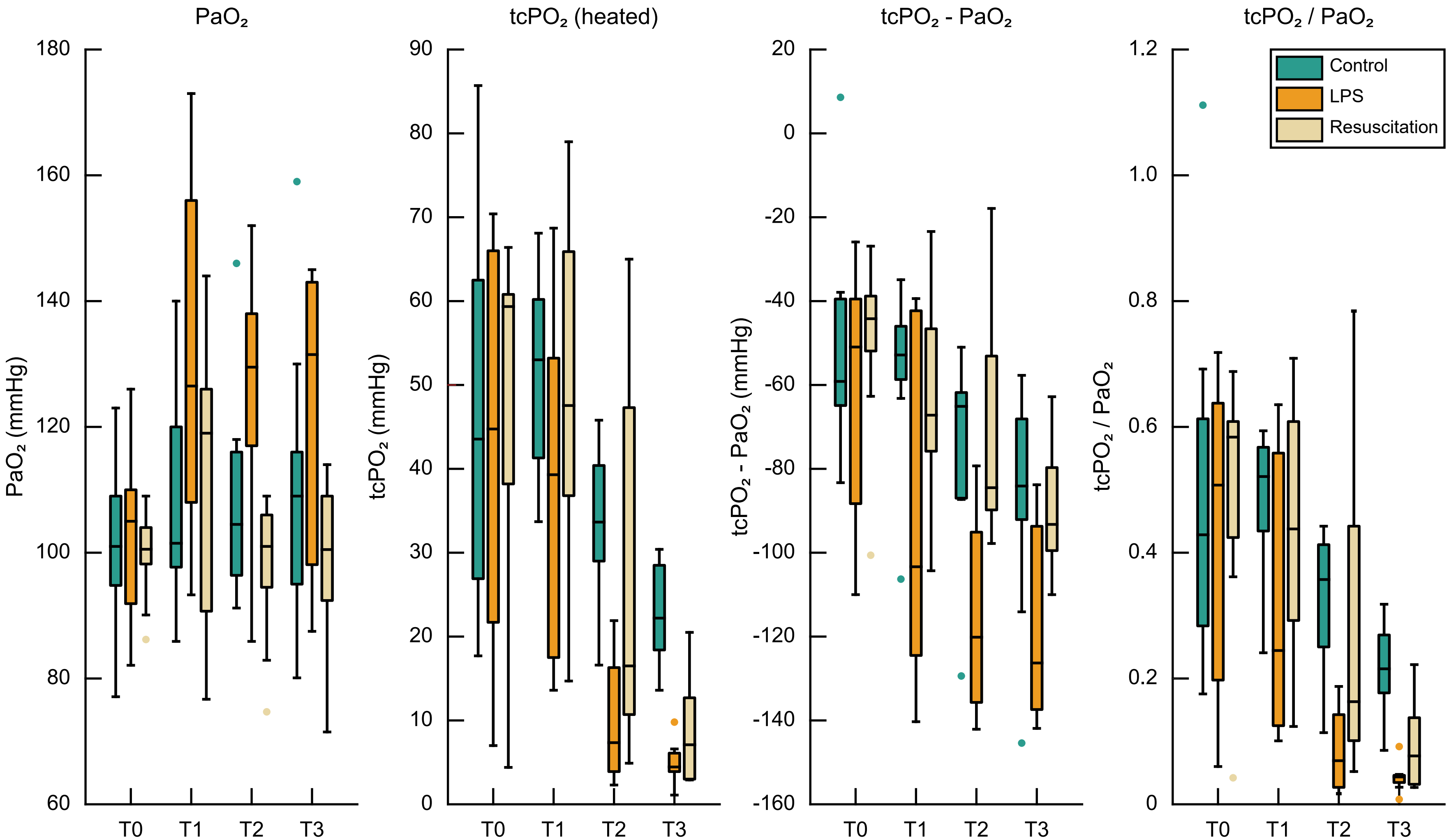

Figure S3 Boxplots of PaO_2, $tcPO_2$, $tcPO_2$ - PaO_2, and $tcPO_2/PaO_2$ per study group. *LPS* lipopolysaccharide, *PaO_2* arterial partial pressure of oxygen, *T* time point, *$tcPO_2$* transcutaneous oxygen.

5 **Supplementary Figure S4** Boxplots MAP and lactate

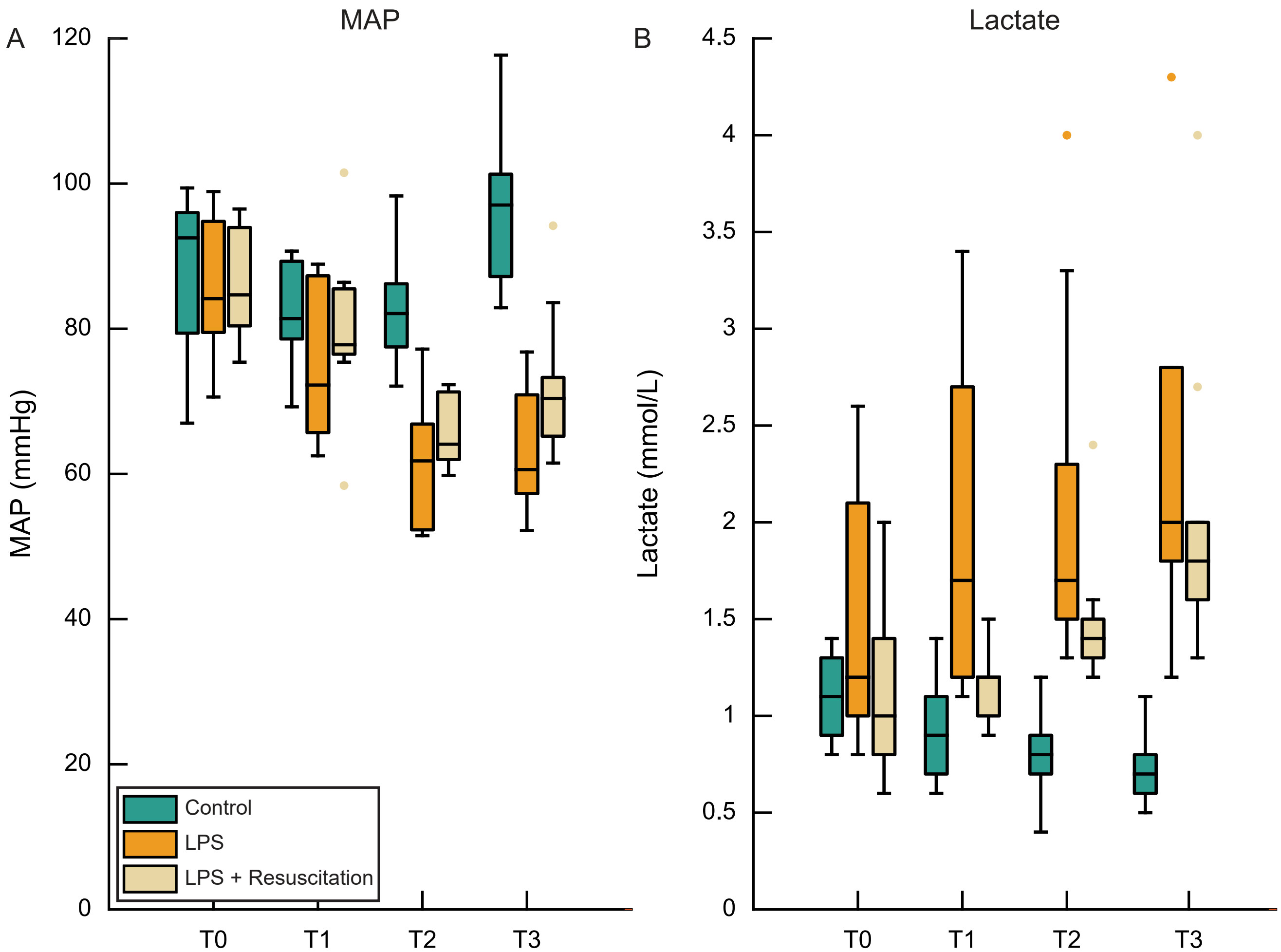

Figure S4 Boxplots of **(A)** MAP and **(B)** lactate per study group. *LPS* lipopolysaccharide, *MAP* mean arterial pressure, *T* time point.

6 **Supplementary Figure S5** Boxplots heating power

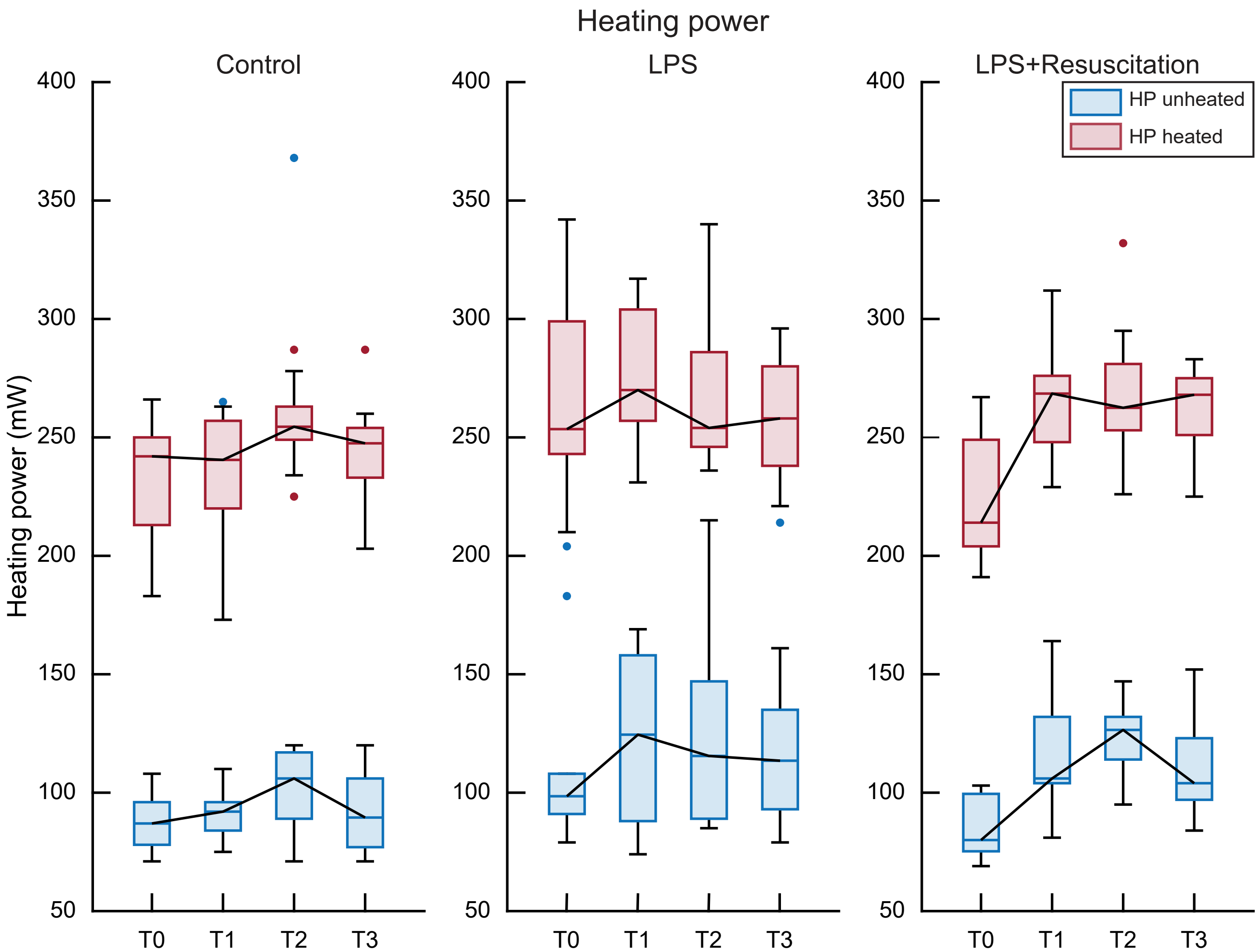

Figure S5 Boxplots of heating power of heated (red) and unheated (blue) transcutaneous measurement per study group. *HP* heating power, *LPS* lipopolysaccharide, *T* time point.

7 **Supplementary Table S2** CO_2 related parameters per study group

**Parameter**	**Time**	**Control (*n*=10)**	**LPS (*n*=10)**	**LPS+Resuscitation (*n*=10)**	***p*-value**
$PaCO_2$ (mmHg)	T0	38.7 [37.1-39.2]	38.0 [37.0-41.2]	39.5 [37.5-42.6]	0.59
	T1	37.2 [34.0-39.4]	41.5 [38.9-45.6]	42.2 [38.6-44.7]	0.03
	T2	37.5 [35.8-41.3]	37.8 [35.0-38.6]	43.0 [41.1-44.7]	$0.01^{\#+}$
	T3	38.6 [34.5-40.2]	37.0 [35.1-40.9]	42.0 [40.3-45.4]	$0.04^{\#}$
	p-value	0.84	0.02	0.49	
$etCO_2$ (mmHg)	T0	45.4 [41.1-48.4]	46.9 [43.7-48.6]	48.8 [46.1-50.4]	0.34
	T1	45.0 [42.0-49.3]	51.8 [50.2-54.8]	51.0 [47.4-53.1]	0.02^{*}
	T2	46.9 [44.2-50.4]	47.2 [43.3-48.8]	49.9 [47.6-53.0]	0.10
	T3	48.4 [42.8-49.3]	46.5 [42.0-47.8]	48.8 [47.6-51.9]	0.17
	p-value	0.62	<0.01	0.79	
$tcPCO_2$ (heated) (mmHg)	T0	42.6 [40.1-44.2]	43.6 [40.8-48.4]	42.9 [41.1-45.6]	0.78
	T1	40.4 [37.4-44.1]	53.2 [46.9-56.3]	48.8 [48.2-52.4]	$<0.01^{*\#}$
	T2	43.0 [40.2-45.7]	54.3 [49.1-59.1]	54.2 [48.5-64.5]	$<0.01^{*\#}$
	T3	48.3 [45.4-49.9]	61.9 [57.2-68.0]	69.2 [63.9-83.0]	$<0.01^{*\#}$
	p-value	0.01^{5}	$<0.01^{3}$	$<0.01^{235}$	
$tcPCO_2$ (unheated) (mmHg)	T0	67.8 [60.8-71.2]	67.6 [62.2-82.5]	66.6 [61.6-69.3]	0.80
	T1	55.5 [47.1-57.5]	73.8 [70.3-82.6]	67.3 [64.6-76.0]	$<0.01^{*\#}$
	T2	49.2 [46.0-51.7]	69.8 [65.2-71.6]	69.3 [61.3-72.0]	$<0.01^{*\#}$
	T3	53.5 [44.9-55.8]	67.4 [60.6-69.6]	65.8 [61.0-70.5]	$0.01^{*\#}$
	p-value	<0.01	0.07	0.90	

CO_2 difference and index

$tcPCO_2$ heated - $PaCO_2$ (mmHg)	T0	4.9 [3.1-6.5]	4.9 [4.3-6.7]	4.3 [2.6-5.9]	0.59
	T1	3.9 [2.9-5.0]	8.6 [6.8-11.7]	9.3 [5.4-11.4]	0.02^{*}
	T2	4.4 [4.2-7.0]	15.8 [13.4-19.5]	10.1 [6.9-21.8]	0.01^{*}
	T3	8.0 [6.2-12.2]	25.0 [21.0-29.4]	27.1 [18.2-44.4]	$<0.01^{*\#}$
	p-value	$<0.01^{5}$	$<0.01^{35}$	$<0.01^{35}$	
$tcPCO_2$ unheated - $PaCO_2$ (mmHg)	T0	30.2 [26.0-35.0]	30.2 [23.3-39.7]	27.4 [19.0-29.8]	0.52
	T1	18.8 [14.7-19.3]	34.1 [24.2-35.4]	27.5 [23.7-35.1]	$0.01^{*\#}$
	T2	15.9 [13.5-21.4]	36.8 [32.4-42.8]	25.6 [20.6-33.0]	$<0.01^{*\#}$
	T3	13.4 [10.6-15.8]	26.5 [21.0-31.7]	23.9 [16.2-29.4]	$<0.01^{*\#}$
	p-value	$<0.01^{123}$	0.10	0.83	
$tcPCO_2$ heated / $PaCO_2$	T0	1.11 [1.08-1.21]	1.13 [1.11-1.18]	1.12 [1.07-1.15]	0.53
	T1	1.10 [1.08-1.13]	1.20 [1.13-1.29]	1.23 [1.12-1.31]	0.07
	T2	1.12 [1.11-1.19]	1.40 [1.35-1.51]	1.24 [1.16-1.50]	0.01^{*}
	T3	1.20 [1.17-1.32]	1.67 [1.58-1.77]	1.64 [1.40-2.00]	$0.01^{*\#}$
	p-value	0.01	$<0.01^{235}$	$<0.01^{35}$	
$tcPCO_2$ unheated / $PaCO_2$	T0	1.82 [1.67-1.94]	1.81 [1.64-1.94]	1.73 [1.48-1.75]	0.25
	T1	1.48 [1.42-1.56]	1.81 [1.49-1.98]	1.76 [1.56-1.83]	0.04
	T2	1.33 [1.22-1.37]	1.77 [1.72-1.90]	1.49 [1.41-1.70]	$<0.01^{*\#+}$
	T3	1.35 [1.33-1.40]	1.71 [1.51-1.84]	1.56 [1.42-1.73]	$<0.01^{*\#}$
	p-value	$<0.01^{1234}$	0.61	0.04	

Supplementary Table S2 Continued

CO_2 difference and index using $etCO_2$

tcPCO_2 heated - etCO_2 (mmHg)	T0	-2.5 [-5.4-1.1]	-2.6 [-3.8--0.8]	-4.2 [-6.5--2.7]	0.52
	T1	-5.7 [-6.6--3.6]	0.9 [-3.0--2.5]	-0.5 [-3.3-0.8]	0.02[*#]
	T2	-5.5 [-6.5--2.8]	8.6 [1.4-11.2]	1.7 [-1.0-11.1]	<0.01[*#]
	T3	-0.9 [-3.6-1.9]	17.3 [13.5-25.0]	19.7 [8.9-38.3]	<0.01[*#]
	p-value	<0.01	<0.01[235]	<0.01[35]	
tcPCO_2 unheated - etCO_2 (mmHg)	T0	25.2 [17.0-29.4]	22.3 [13.9-32.1]	17.7 [14.8-22.8]	0.65
	T1	9.3 [7.5-12.1]	22.1 [16.3-28.7]	21.2 [11.1-24.4]	0.01[*]
	T2	3.7 [2.9-4.2]	22.1 [18.3-24.1]	15.6 [11.9-20.1]	<0.01[*#]
	T3	4.0 [3.1-4.9]	18.9 [16.4-24.4]	19.2 [8.4-21.8]	<0.01[*#]
	p-value	<0.01	0.49	0.47	
etCO_2 / tcPCO_2 heated	T0	0.95 [0.89-1.03]	0.95 [0.9-0.97]	0.92 [0.86-0.93]	0.40
	T1	0.87 [0.85-0.92]	1.02 [0.95-1.04]	0.99 [0.94-1.02]	0.01[#]
	T2	0.89 [0.87-0.94]	1.20 [1.03-1.22]	1.03 [0.98-1.23]	<0.01[*#]
	T3	0.99 [0.91-1.04]	1.37 [1.31-1.55]	1.40 [1.18-1.83]	<0.01[*#]
	p-value	0.01	<0.01[235]	<0.01[35]	
etCO_2 / tcPCO_2 unheated	T0	1.56 [1.37-1.71]	1.48 [1.33-1.63]	1.32 [1.26-1.42]	0.27
	T1	1.22 [1.14-1.28]	1.41 [1.29-1.57]	1.45 [1.21-1.52]	0.03[*]
	T2	1.07 [1.04-1.09]	1.46 [1.41-1.50]	1.31 [1.23-1.38]	<0.01[*#]
	T3	1.10 [1.06-1.12]	1.43 [1.32-1.53]	1.39 [1.19-1.45]	0.01[*#]
	p-value	<0.01[123]	0.39	0.59	

Unheated - heated tcPCO_2 difference and index

tcPCO_2 unheated - tcPCO_2 heated (mmHg)	T0	24.2 [20.5-28.2]	22.1 [18.8-33.2]	23.0 [16.9-23.9]	0.60
	T1	14.2 [8.9-15.1]	22.4 [16.8-25.9]	16.8 [12.0-23.0]	0.02[*]
	T2	8.5 [4.1-10.3]	13.9 [9.1-17.6]	8.8 [3.9-14.9]	0.27
	T3	6.5 [3.1-9.8]	0.7 [-8.3-8.8]	-1.2 [-6.6-2.0]	0.11
	p-value	<0.01[123]	<0.01[235]	<0.01[235]	
tcPCO_2 unheated / tcPCO_2 heated	T0	1.59 [1.46-1.67]	1.51 [1.42-1.65]	1.52 [1.35-1.56]	0.28
	T1	1.31 [1.24-1.38]	1.42 [1.32-1.51]	1.34 [1.26-1.49]	0.24
	T2	1.16 [1.02-1.21]	1.25 [1.15-1.32]	1.19 [1.07-1.28]	0.27
	T3	1.16 [1.06-1.21]	1.01 [0.88-1.15]	0.98 [0.93-1.03]	0.07
	p-value	<0.01[12345]	<0.01[2345]	<0.01[235]	

etCO_2 end-tidal CO_2, *LPS* lipopolysaccharide, *n* number, *PaCO_2* arterial partial pressure of carbon dioxide, *T* time point, *tcPCO_2* transcutaneous carbon dioxide.

[*] Significant difference between Control and LPS group

[#] Significant difference between Control and LPS with fluid resuscitation group

[+] Significant difference between LPS and LPS with fluid resuscitation group

[1] Significant difference between T0 and T1

[2] Significant difference between T0 and T2

[3] Significant difference between T0 and T3

[4] Significant difference between T1 and T2

[5] Significant difference between T1 and T3

8 **Supplementary Table S3** O_2 related parameters per study group

Parameter	Time	Control (*n*=10)	LPS (*n*=10)	LPS+Resuscitation (*n*=10)	*p*-value
PaO_2 (mmHg)	T0	101.0 [95.8-107.0]	105.0 [92.5-110.0]	101.0 [98.2-104.0]	0.81
	T1	102.0 [98.3-118.0]	126.0 [108.0-155.0]	119.0 [94.8-124.0]	0.21
	T2	104.0 [97.1-115.0]	130.0 [118.0-138.0]	101.0 [95.6-105.0]	0.01[+]
	T3	109.0 [97.2-115.0]	132.0 [100.0-142.0]	100.0 [92.4-108.0]	0.12
	p-value	0.61	0.06	0.37	
SpO_2 (%)	T0	96 [94-97]	96 [94-96]	95 [93-96]	0.62
	T1	96 [95-96]	96 [93-97]	96 [94-96]	0.98
	T2	96 [94-96]	96 [95-98]	96 [94-97]	0.80
	T3	96 [95-96]	96 [94-98]	95 [94-96]	0.43
	p-value	0.91	0.81	0.79	
FiO_2 (%)	T0	29 [27-30]	33 [28-38]	30 [30-34]	0.25
	T1	33 [28-35]	40 [40-45]	44 [40-45]	<0.01[*#]
	T2	35 [30-35]	40 [38-43]	38 [35-40]	0.01[*]
	T3	35 [30-36]	40 [36-44]	40 [38-44]	0.03
	p-value	0.02	<0.01[1]	<0.01[123]	
$tcPO_2$ (heated) (mmHg)	T0	43.5 [28.7-58.5]	44.8 [26.7-63.4]	59.3 [40.7-60.7]	0.83
	T1	53.0 [42.0-59.2]	39.3 [18.3-51.0]	47.5 [37.0-63.7]	0.40
	T2	33.6 [29.4-39.5]	7.3 [4.3-15.7]	16.5 [10.8-42.9]	<0.01[*]
	T3	22.2 [18.7-27.4]	4.4 [4.0-5.9]	7.1 [3.4-12.1]	<0.01[*#]
	p-value	<0.01[345]	<0.01[2345]	<0.01[35]	

PO_2 difference and index

$tcPO_2$ heated - PaO_2 (mmHg)	T0	-59.2 [-64.6--42.9]	-51.0 [-81.0--39.8]	-44.2 [-51.5--39.4]	0.56
	T1	-52.8 [-58.5--46.5]	-103.0 [-122.0--49.9]	-67.2 [-75.2--51.0]	0.11
	T2	-65.1 [-84.7--62.0]	-120.0 [-133.0--101.0]	-84.5 [-89.8--54.8]	<0.01[*+]
	T3	-84.1 [-90.6--71.4]	-126.0 [-136.0--94.6]	-93.2 [-98.9--82.0]	0.02[*]
	p-value	<0.01[35]	<0.01[23]	0.03[3]	
$tcPO_2$ heated / PaO_2	T0	0.43 [0.30-0.57]	0.51 [0.25-0.62]	0.58 [0.44-0.61]	0.75
	T1	0.52 [0.44-0.56]	0.24 [0.15-0.49]	0.44 [0.31-0.59]	0.21
	T2	0.36 [0.25-0.40]	0.07 [0.03-0.13]	0.16 [0.10-0.43]	<0.01[*+]
	T3	0.22 [0.18-0.27]	0.04 [0.04-0.05]	0.08 [0.03-0.13]	<0.01[*#]
	p-value	<0.01[345]	<0.01[2345]	<0.01[35]	

FiO_2 fraction of inspired oxygen, *LPS* lipopolysaccharide, *n* number, *PaO_2* arterial partial pressure of oxygen, *SpO_2* peripheral oxygen saturation, *T* time point, *$tcPO_2$* transcutaneous oxygen.

[*] Significant difference between Control and LPS group

[#] Significant difference between Control and LPS with fluid resuscitation group

[+] Significant difference between LPS and LPS with fluid resuscitation group

[1] Significant difference between T0 and T1

[2] Significant difference between T0 and T2

[3] Significant difference between T0 and T3

[4] Significant difference between T1 and T2

[5] Significant difference between T1 and T3

9 **Supplementary Table S4** $TcPO_2$ / $TcPCO_2$ and heating power per study group

**Parameter**	**Time**	**Control (*n*=10)**	**LPS (*n*=10)**	**LPS+Resuscitation (*n*=10)**	***p*-value**
$tcPO_2$ / $tcPCO_2$ (heated)	T0	0.43 [0.30-0.57]	0.51 [0.25-0.62]	0.58 [0.44-0.61]	0.78
	T1	0.52 [0.44-0.56]	0.24 [0.15-0.49]	0.44 [0.31-0.59]	0.03[*]
	T2	0.36 [0.25-0.40]	0.07 [0.03-0.13]	0.16 [0.10-0.43]	<0.01[*]
	T3	0.22 [0.18-0.27]	0.04 [0.04-0.05]	0.08 [0.03-0.13]	<0.01[*#]
	p-value	<0.01[345]	<0.01[2345]	<0.01[35]	
Heating power (heated) (mW)	T0	242 [218-250]	254 [245-291]	214 [204-248]	0.04[+]
	T1	240 [224-254]	270 [258-299]	268 [252-275]	0.01[#]
	T2	254 [250-263]	254 [246-285]	262 [253-279]	0.71
	T3	248 [234-254]	258 [240-279]	268 [254-274]	0.15
	p-value	0.05	0.70	0.04[123]	
Heating power (unheated) (mW)	T0	87 [79-95]	98 [93-106]	80 [76-99]	0.11
	T1	92 [84-96]	124 [88-155]	106 [104-126]	0.20
	T2	106 [89-117]	116 [91-143]	126 [114-132]	0.23
	T3	90 [77-106]	114 [93-134]	104 [98-120]	0.10
	p-value	0.17	0.60	<0.01[12]	

LPS lipopolysaccharide, *n* number, *T* time point, $tcPCO_2$ transcutaneous carbon dioxide, $tcPO_2$ transcutaneous oxygen.

[#] Significant difference between Control and LPS with fluid resuscitation group
[1] Significant difference between T0 and T1
[2] Significant difference between T0 and T2
[3] Significant difference between T0 and T3
[4] Significant difference between T1 and T2
[5] Significant difference between T1 and T3

10 Supplementary Table S5 MAP and lactate per study group

**Parameter**	**Time**	**Control (*n*=10)**	**LPS (*n*=10)**	**LPS+Resuscitation (*n*=10)**	***p*-value**
MAP (mmHg)	T0	92.5 [82.1-95.5]	84.2 [80.3-92.4]	84.7 [80.9-92.0]	0.74
	T1	81.4 [79.0-87.6]	72.2 [66.2-84.0]	77.8 [76.8-84.9]	0.15
	T2	82.1 [78.1-85.5]	61.8 [52.5-64.0]	64.1 [62.2-69.8]	<0.01[*,#]
	T3	97.0 [88.0-101.0]	60.6 [57.6-70.2]	70.4 [66.2-73.0]	<0.01[*,#]
	p-value	0.01[5]	<0.01[2,3]	<0.01[3,5]	
Lactate (mmol/L)	T0	1.1 [0.9-1.3]	1.2 [1.0-2.0]	1.0 [0.8-1.4]	0.33
	T1	0.9 [0.7-1.1]	1.7 [1.2-2.7]	1.2 [1.0-1.2]	<0.01[*,+]
	T2	0.8 [0.7-0.9]	1.7 [1.5-2.2]	1.4 [1.3-1.5]	<0.01[*,#]
	T3	0.7 [0.6-0.8]	2.0 [1.8-2.7]	1.8 [1.6-2.0]	<0.01
	p-value	<0.01[3]	0.23	<0.01[3,5]	

LPS lipopolysaccharide, *MAP* mean arterial pressure, *n* number, *T* time point.

[*] Significant difference between Control and LPS group
[#] Significant difference between Control and LPS with fluid resuscitation group
[+] Significant difference between LPS and LPS with fluid resuscitation group
[1] Significant difference between T0 and T1
[2] Significant difference between T0 and T2
[3] Significant difference between T0 and T3
[4] Significant difference between T1 and T2
[5] Significant difference between T1 and T3
